# Combining controlled-release urea with potassium chloride to reduce soil N/K leaching and promote growth of Italian ryegrass

**DOI:** 10.1038/s41598-023-27620-5

**Published:** 2023-01-06

**Authors:** Jibiao Geng, Xiuyi Yang, Shutong Lei, Qingping Zhang, Hui Li, Ying Lang, Xianqi Huo, Qianjin Liu

**Affiliations:** Shandong Provincial Key Laboratory of Water and Soil Conservation and Environmental Protection, College of Agriculture and Forestry Science/Resources and Environment, Linyi University, Linyi, 276000 Shandong China

**Keywords:** Physiology, Environmental sciences

## Abstract

Nitrogen (N) and potassium (K) are essential nutrients for Italian ryegrass (*Lolium multiflorum* L.) growth. A 2-year field experiment with a split-plot design was conducted to study the effect of N fertilizer type combined with different K fertilizer rates on the soil mineral N and K availability, and growth characteristics of Italian ryegrass. The main plots were assigned to two N fertilizer types, controlled-release urea (CRU) and common urea. While low, moderate and high potassium chloride (KCl) rates (150, 300 and 450 kg ha^−1^, respectively) were assigned to the subplots. Compared with the common urea treatments, the CRU significantly increased the SPAD value, plant height, leaf area, and photosynthetic index of Italian ryegrass, which significantly prolonged the green stage of Italian ryegrass and prevented premature senescence. Moreover, the dry yields of the CRU increased by 4.5–12.5% in 2019 and 10.9–25.3% in 2020 compared with the urea, respectively. At the same time, compared with the KCl150 and KCl450 treatments, the KCl300 treatment resulted in better plant growth. Overall, the CRU × KCl300 maximized the soil inorganic N and different soil K forms, and reduced the soil N/K leaching. The root length, volume, surface area, average diameter, tips and branches were also improved, and there was a significant N × K interaction effect on the tips. The CRU combined with 300 kg ha^−1^ KCl fertilization enhanced crop growth by improving leaf photosynthesis, soil fertility, and yield and should be recommended as the best fertilizer ratio for Italian ryegrass production.

## Introduction

Grassland accounts for 41.7% of China’s total land area^1^. For a long time, as the main means of production and material basis, grasslands have provided an important contribution to the development of animal husbandry. Grasslands have an important ecological function, which plays a central role in water and soil conservation, air purification, climate regulation and biodiversity conservation^2^. Italian ryegrass (*Lolium multiflorum* L.) is a globally cultivated grass. This grass has many tillers at its roots, grows quickly and has good grazing resistance. Italian ryegrass has a strong adaptability and high yield and nutrition, which play a role in improving soil fertility^3,4^. Italian ryegrass is widely used at parks, golf courses and other recreational sporting areas as the first choice of lawn grass in foreign countries^5^. Various production technologies and late management measures have also become new research topics. At present, research on ryegrass in lawns, playgrounds and other fields in China is at an immature stage. A large number of researchers have focused on management technology after ryegrass planting^6,7^.

N is not only the basis of forage genetic material but also the composition of many important organic compounds^8^. It is very important for life cycle activities, yield and nutritional quality of forage. Italian ryegrass does not have the ability to fix N, so N in the soil is the key factor in grass growth and development^9^. The application of N fertilizer can significantly improve the yield of Italian ryegrass, and the plant can absorb both ammonium and nitrate N^10^. Moreover, the Italian ryegrass is a fast-growing forage grass with a high N requirement and, therefore, strongly relies on soil N content to maintain adequate forage yield^11^.

The utilization rate of fertilizer in China is generally low, which is mainly due to the rapid dissolution of instant fertilizer after it is applied to the soil^12^. The crop cannot absorb and utilize N in time, which results in the loss of most of the N in gaseous or water-soluble forms and causes a series of environmental problems. Such as eutrophication of surface water, nitrate pollution of groundwater and agricultural products, and ammonia and N oxides emitted to the ozone layer^13^. In addition, it is difficult to apply fertilizer to Italian ryegrass every time after cutting. Thus, reducing the times of applying fertilizer and improving the yield and quality of Italian ryegrass are the key problems to address in the planting process^14^. It is an effective way to develop a new type of slow-release fertilizer to meet the needs of crop growth.

In recent years, controlled-release urea (CRU) has been widely used worldwide^15–17^. CRU can release N slowly in the form of a resin polymer coating, which continuously supply nutrients needed for ryegrass growth. Moreover, CRU can decrease the number of topdressings and labour intensity in the later stage, which simplify cultivation technology, save time and labour, and reduce environmental pollution^18^.

KCl is commonly used in agricultural production. It has a low price and high nutrient content, and the application of appropriate amounts can promote the growth of Italian ryegrass^19^. In addition, K can improve the photosynthetic capacity and disease resistance of plants and then extend the green period^20^. In addition, a single application of N and K or mixed application of different proportions can increase the yield of Italian ryegrass, but the effect of a mixed application is better than a single application^21^. According to the supply and demand curve of soil for nutrient elements, the most appropriate fertilization amount and fertilization type can be formulated.

N can improve photosynthesis, and thus dry matter production^22^. While, K has a major importance in root growth and disease resistance^23^. The possible interactions between the two nutrients as they have not enough been studied in Italian Ryegrass. In previous studies on the effect of N × K fertilizer application on crop growth, the positive interaction of N and K reduced the cost of fertilizer and contributed to food security^24,25^. N fertilizer application increases the plant K absorption efficiency and K fertilizer application also solved the problem of N pollution by inducing high N absorption efficiency of crops^26^. Eventually, the mutual promotion of N and K enhance the yield and quality of crops^24^. It is a feasible way to increase the input of K fertilizer and improve the efficiency of N utilization. Understanding the mechanism of N × K interaction is vital to guide the best practice of nutrient management in agriculture production.

Furthermore, there are few reports on the interactive effects of CRU combined with KCl on Italian ryegrass growth. Hence, the objective of this study was to investigate the effects of CRU in combination with KCl on (i) soil inorganic N (NO_3_^−^-N and NH_4_^+^-N), (ii) soil K forms (available K, water-soluble K, exchangeable K and non-exchangeable K), (iii) growth and photosynthetic characteristics, and (iv) yield and fertilizer use efficiency of Italian ryegrass.

## Results

### Release characteristics of the CRU

**Figure 1:**
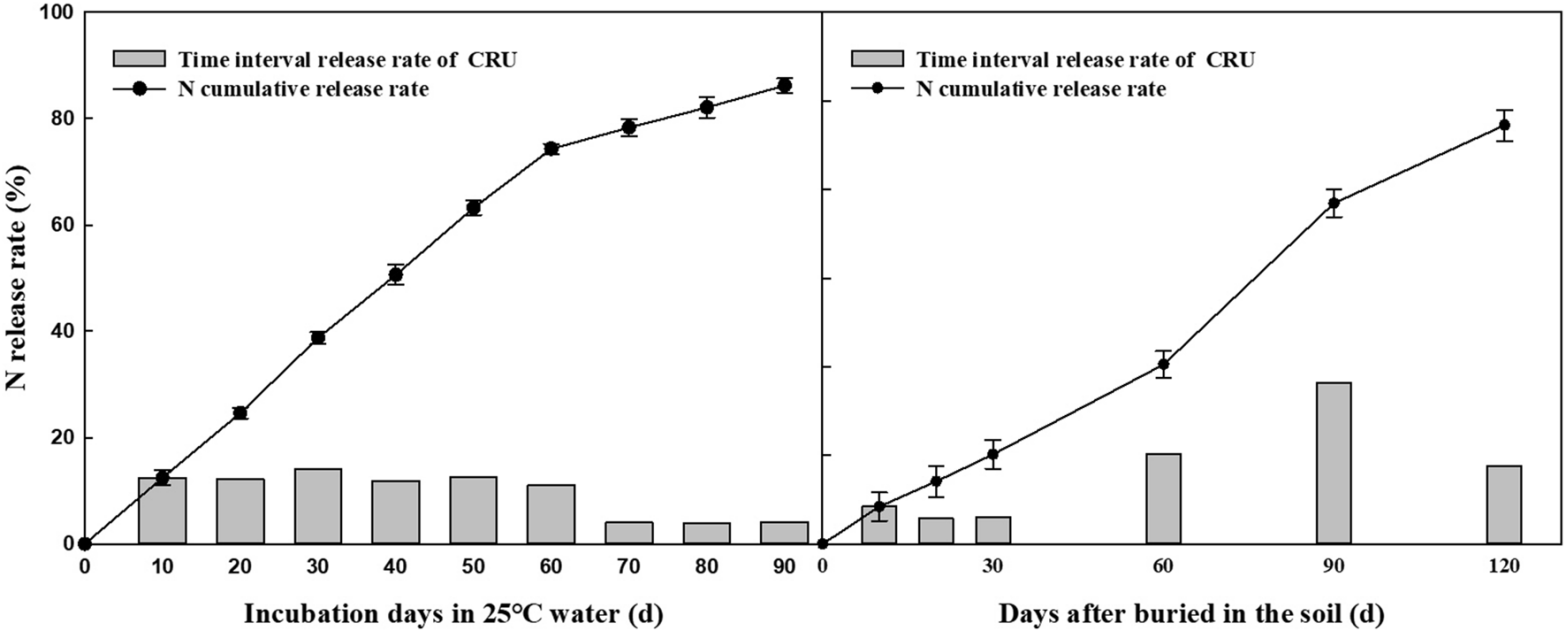
Release of N from CRU.

The release characteristics of the CRU in the soil were released in the form of “S”, reaching 80% in approximately 100 days (Fig. 1). The release period of the CRU in the soil was almost 120 days, the release rate was slow within 0 ~ 60 day, the release rate increased within 60 ~ 90 day, and the nutrient decline period was 90 ~ 120 day, which met the N demand of Italian ryegrass.

### Soil inorganic N and K form

**Figure 2:**
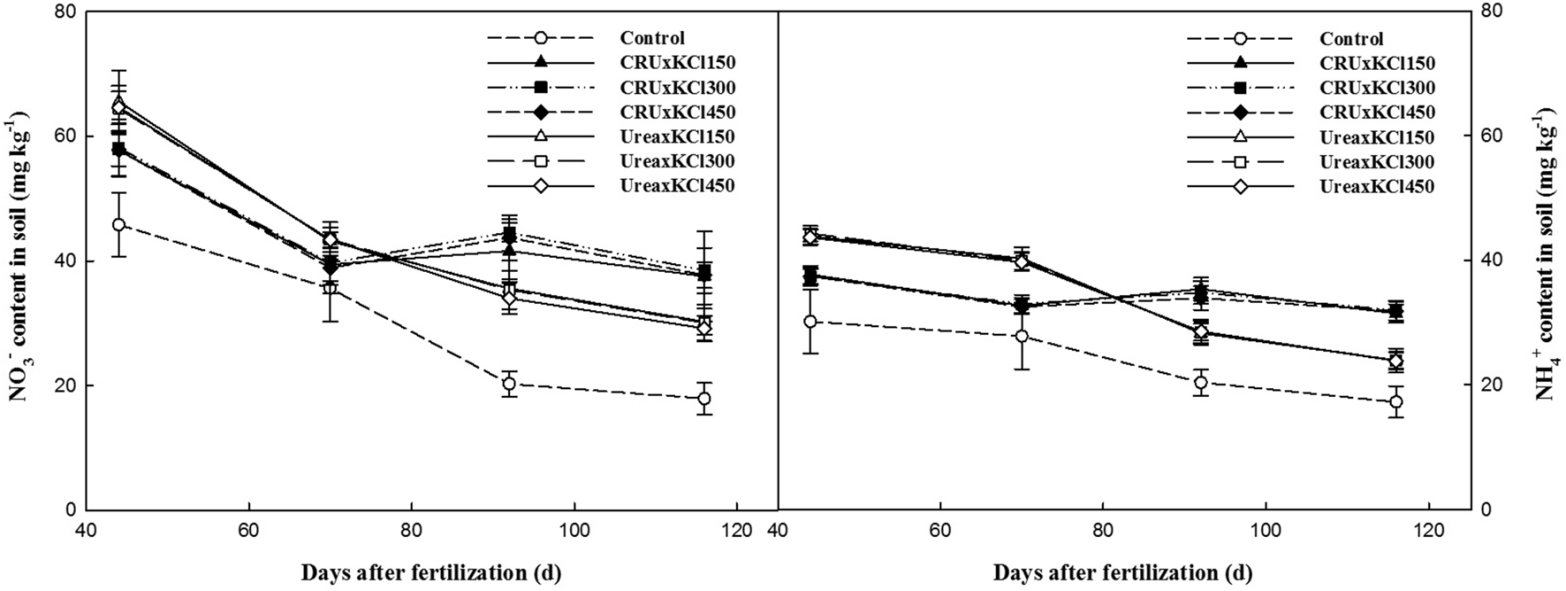
Changes in the soil NO_3_^−^-N and NH_4_^+^-N contents.

The contents of NO_3_^−^-N and NH_4_^+^-N in the soil in the control treatment were the lowest among all the treatments in the whole plant growing season (Fig. 2). With the increase in the KCl application rate, the contents of NO_3_^−^-N and NH_4_^+^-N in the soil changed little, which was not related to the type of N application. However, at the early stage of growth, the contents of NO_3_^−^-N and NH_4_^+^-N in the urea treatment were higher than those in the CRU treatment, but after the second clipping, the contents of NO_3_^−^-N and NH_4_^+^-N in the urea treatment decreased rapidly; in addition, the contents of NO_3_^−^-N and NH_4_^+^-N in the urea treatment were lower than those in the CRU treatment.

**Figure 3:**
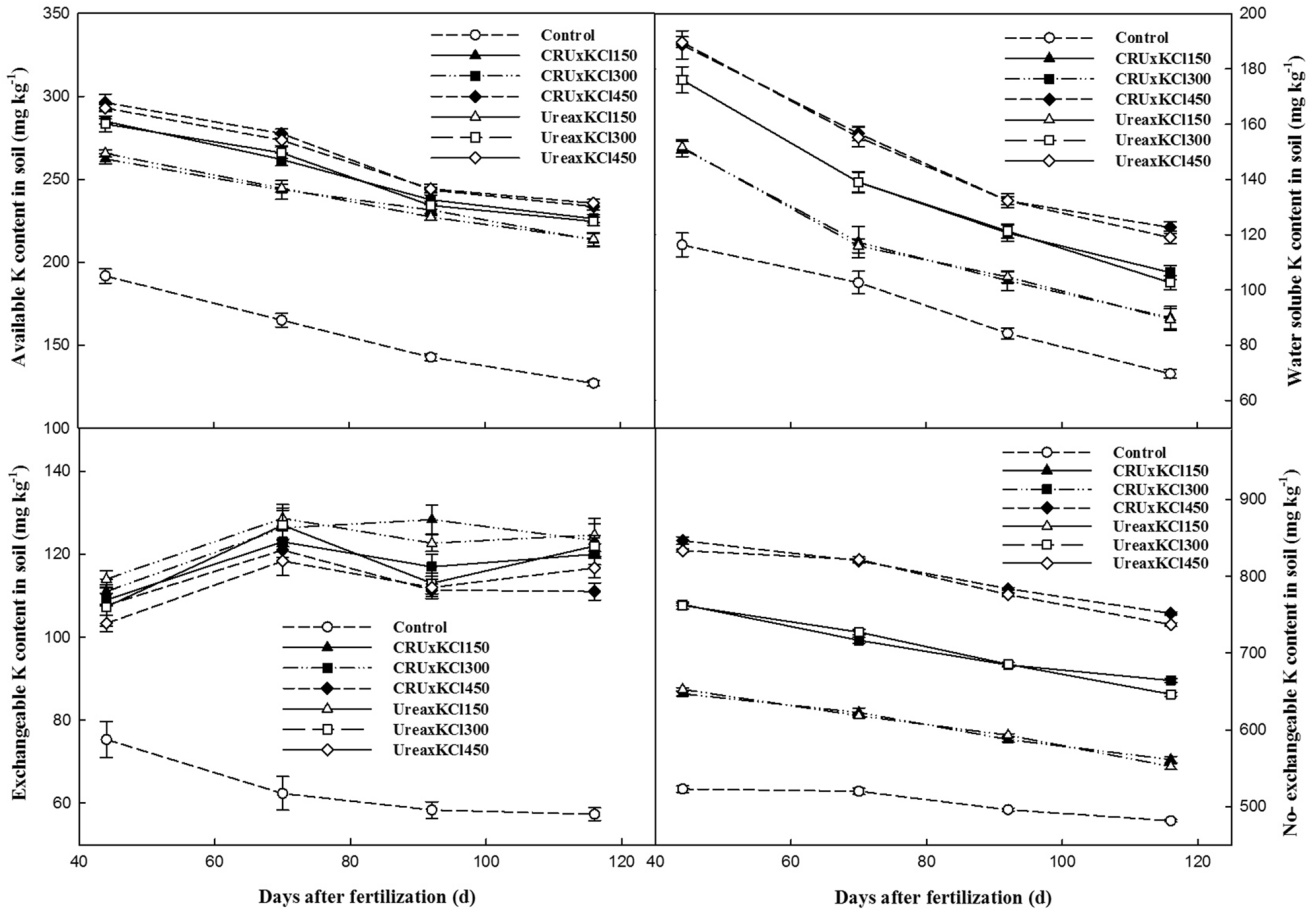
Changes in the soil K forms.

Overall, the contents of available K, water soluble K, exchangeable K and non-exchangeable K were significantly affected by the KCl application rate. And of the treatments, the control treatment had the lowest values in different developmental stages (Fig. 3). The contents of soil available K, water soluble K and non-exchangeable K decreased gradually, but exchangeable K showed a fluctuating trend. Regardless of which type of N fertilizer was combined, the content of soil available K, water soluble K and exchangeable K improved with the increase in the KCl application rate, and there was a significant difference between the different K fertilizer treatments.

### Plant height and leaf area

In the whole growth period of Italian ryegrass, the plant heights of the different fertilization treatments increased first and then decreased (Table 1). There was no significant difference between the different fertilization treatments at the first and second clipping stages, but the advantage of the CRU treatment group was obvious; in addition, the average value was higher than that of the control treatment. During the mowing period of the third and fourth clippings, Italian ryegrass growth was in the transition period from maturity to senescence, the plant demand for nutrients decreased, and the ability to absorb fertilizer also decreased. Under the condition of applying the same amount of N, the plants in the KCl150 and the KCl300 treatments were taller, and those in the KCl450 treatment were shorter. At the same time, the plant height of the CRU treatment group was significantly higher than that of the urea treatment groups.

**Table 1:** The plant height of Italian ryegrass.

Treatment	First clipping (cm)	Second clipping (cm)	Third clipping (cm)	Fourth clipping (cm)
**N fertilizer type**				
CRU	17.64 a	25.22 a	24.47 a	15.50 a
Urea	16.67 a	23.94 a	19.83 b	8.06 b
**K fertilizer rate (kg ha**^**−1**^**)**				
150	17.78 a	23.47 a	22.70 a	11.17 b
300	16.48 a	25.17 a	21.62 a	13.08 a
450	17.20 a	25.12 a	20.63 a	11.08 b
**N × K interaction**				
Control	16.27 a	21.43 c	20.30 b	8.32 b
CRU × KCl150	17.83 a	24.57 abc	25.23 a	14.33 a
CRU × KCl300	16.77 a	25.90 a	23.23 ab	17.02 a
CRU × KCl450	18.33 a	25.20 ab	21.93 ab	15.16 a
Urea × KCl150	17.73 a	22.36 bc	20.17 b	8.32 b
Urea × KCl300	16.20 a	24.43 abc	20.00 b	9.16 b
Urea × KCl450	16.07 a	25.03 ab	19.33 b	7.03 b
**Source of variance**				
N	0.1619	0.1735	0.0023	< 0.0001
K	0.3002	0.2415	0.1858	0.0632
N × K	0.3855	0.6337	0.4809	0.5067

Means followed by different lowercase letters in the same column were significantly different based on analyses with ANOVAs followed by Duncan tests (*P* < 0.05).

*Control* no N and K fertilizer, *CRU* controlled-release urea, *KCl* potassium chloride.

Similarly, the leaf area of Italian ryegrass in the whole growth period increased first and then decreased with the growth of the plant (Table 2). At the second clipping period, the apparent leaf area increased the fastest. The leaves of the plants were long and thin, and there was no difference between the fertilization treatments at the first and second clipping stages, but fertilization was more suitable to its growth; in addition, the leaf area of the plants with fertilization was relatively large. During the third and fourth clipping stages, in comparison with the control and common urea treatments, the CRU treatments resulted in a significant difference, which indicated that the CRU could delay plant senescence to some extent. In addition, the effect of N fertilizer on ryegrass leaf area was greater than that of KCl fertilizer, and the sustainability of combined application is more important. There was no significant N × K interaction effect on the plant height and leaf area (except at the third clipping).

**Table 2:** The leaf area of Italian ryegrass.

Treatment	First clipping (mm^2^)	Second clipping (mm^2^)	Third clipping (mm^2^)	Fourth clipping (mm^2^)
**N fertilizer type**				
CRU	295.72 a	542.99 a	475.30 a	171.64 a
Urea	282.67 a	614.73 a	389.57 b	57.16 b
**K fertilizer rate (kg ha**^**−1**^**)**				
150	287.10 a	522.52 a	396.50 a	111.93 a
300	286.22 a	593.90 a	418.97 a	135.10 a
450	294.27 a	620.17 a	481.83 a	96.17 b
**N × K interaction**				
Control	328.73 a	491.52 a	330.53 b	108.32 b
CRU × KCl150	289.40 a	417.61 a	381.93 b	167.86 a
CRU × KCl300	305.87 a	589.44 a	455.47 ab	184.50 a
CRU × KCl450	291.92 a	622.02 a	588.53 a	162.63 a
Urea × KCl150	284.85 a	627.56 a	411.12 b	56.07 cd
Urea × KCl300	266.57 a	598.41 a	382.47 b	85.73 bc
Urea × KCl450	296.63 a	618.39 a	375.13 b	29.76 d
**Source of variance**				
N	0.6001	0.3835	0.0319	< 0.0001
K	0.9588	0.5909	0.1536	0.0621
N × K	0.7394	0.4862	0.0484	0.4916

Means followed by different lowercase letters in the same column were significantly different based on analyses with ANOVAs followed by Duncan tests (*P* < 0.05).

*Control* no N and K fertilizer, *CRU* controlled-release urea, *KCl* potassium chloride.

### Leaf SPAD and photosynthetic index

The effect of different fertilization treatments on the SPAD value of Italian ryegrass was different (Table 3). At the first clipping stage, the SPAD value of the CRU × KCl300 treatment was the highest, which was 17.1% higher than that of the control treatment. During the whole growth period of Italian ryegrass, the SPAD value increased first and then decreased, and the SPAD value of the CRU treatment was higher than that of the common urea treatment. Under the three levels of KCl fertilizer, the SPAD value of the KCl150 treatment in the whole growth period was low, which indicates that the insufficient use of K had a certain impact on the SPAD value. The SPAD value of the CRU × KCl300 treatment was the highest at the third and fourth stage, while the effect of N on the SPAD value of ryegrass was greater at the later stage. In comparison with common urea, the application of CRU had a greater advantage in improving the SPAD value. There was no significant N × K interaction effect on the SPAD value (except at the second clipping).

**Table 3:** The leaf SPAD value of Italian ryegrass.

Treatment	First clipping	Second clipping	Third clipping	Fourth clipping
**N fertilizer type**				
CRU	49.76 a	54.70 a	27.49 a	17.44 a
Urea	44.37 b	57.66 b	20.92 b	10.39 b
**K fertilizer rate (kg ha**^**−1**^**)**				
150	45.72 b	54.83 a	24.17 a	14.33 a
300	46.47 ab	57.73 a	25.97 a	15.02 a
450	49.00 a	55.97 a	22.48 a	12.40 a
**N × K interaction**				
Control	45.67 c	50.93 c	25.97 ab	13.66 b
CRU × KCl150	50.00 ab	51.66 c	27.56 ab	14.86 ab
CRU × KCl300	47.66 bc	59.62 a	29.70 a	22.43 a
CRU × KCl450	51.60 a	52.74 bc	25.20 ab	15.03 ab
Urea × KCl150	41.43 d	58.02 ab	20.76 ab	13.80 b
Urea × KCl300	45.27 c	55.80 abc	22.23 ab	7.60 b
Urea × KCl450	46.40 bc	59.16 a	19.76 b	9.77 b
**Source of variance**				
N	0.0008	0.0956	0.002	0.0047
K	0.0746	0.3603	0.2125	0.507
N × K	0.1102	0.0436	0.8487	0.039

Means followed by different lowercase letters in the same column were significantly different based on analyses with ANOVAs followed by Duncan tests (*P* < 0.05).

*Control* no N and K fertilizer, *CRU* controlled-release urea, *KCl* potassium chloride.

The N fertilizer types and KCl rates affected the photosynthesis indicators, but there was no significant difference between their interaction effects (Table 4). In comparison to the urea treatments, the CRU treatment improved the net photosynthetic rate (*P*_n_), stomatal conductance (*G*_s_) and transpiration rate (*T*_r_) and lowered the intercellular carbon dioxide concentration (*C*_i_). In addition, the photosynthesis indicators increased with increasing KCl rates. There was no significant N × K interaction effect on the photosynthesis indicators (except *T*_r_), and of the treatments, the CRU × KCl300 treatment performed the best in terms of Italian ryegrass leaf photosynthesis.

**Table 4:** The leaf photosynthesis chlorophyll parameters of Italian ryegrass at the second clipping stage.

Treatment	*P*_n_ (umol m^−2^ s^−1^)	*G*_s_ (umol mol^−1^)	*T*_r_ (umol m^−2^ s^−1^)	*C*_i_ (umol m^−2^ s^−1^)
**N fertilizer type**				
CRU	295.72 a	542.99 a	475.30 a	171.64 a
Urea	282.67 a	614.73 a	389.57 b	57.16 b
**K fertilizer rate (kg ha**^**−1**^**)**				
150	287.10 a	522.52 a	396.50 a	111.93 a
300	286.22 a	593.90 a	418.97 a	135.10 a
450	294.27 a	620.17 a	481.83 a	96.17 b
**N × K interaction**				
Control	328.73 a	491.52 a	330.53 b	108.32 b
CRU × KCl150	289.40 a	417.61 a	381.93 b	167.86 a
CRU × KCl300	305.87 a	589.44 a	455.47 ab	184.50 a
CRU × KCl450	291.92 a	622.02 a	588.53 a	162.63 a
Urea × KCl150	284.85 a	627.56 a	411.12 b	56.07 cd
Urea × KCl300	266.57 a	598.41 a	382.47 b	85.73 bc
Urea × KCl450	296.63 a	618.39 a	375.13 b	29.76 d
**Source of variance**				
N	0.6001	0.3835	0.0319	< 0.0001
K	0.9588	0.5909	0.1536	0.0621
N × K	0.7394	0.4862	0.0484	0.4916

Means followed by different lowercase letters in the same column were significantly different based on analyses with ANOVAs followed by Duncan tests (*P* < 0.05).

*Control* no N and K fertilizer, *CRU* controlled-release urea, *KCl* potassium chloride, *P*_*n*_ photosynthetic parameters including net photosynthetic rate, *G*_*s*_ stomatal conductance, *C*_*i*_ intercellular carbon dioxide concentration, *T*_*r*_ transpiration rate.

### Root morphology

The N fertilizer types and K fertilizer rates significantly affected the root morphology (total length, surface area, average diameter, root volume, tips and branches) (Table 5). Concretely, the CRU treatments increased the root length, surface area, average diameter, root volume, tips and branches compared with the urea treatments. Besides, the moderate KCl rate treatments markedly improved the root morphology than the low and high KCl rate treatments despite of any N fertilizer type. However, there was no significant N × K interaction effect (except the tips), and of the treatments, the CRU × KCl300 treatment performed the best in terms of Italian ryegrass root growth.

**Table 5:** The root morphology of Italian ryegrass at the fourth clipping stage.

Treatments	Total length (cm)	Surface area (cm^2^)	Average diameter (mm)	Root volume (cm^3^)	Tips	Branches
**N fertilizer type**						
CRU	1480.89 a	180.23 a	0.35 a	1.61 a	14,956 12 a	16,076.37 a
Urea	1377.67 b	159.56 b	0.32 b	1.49 b	13,947 .33 b	14,872.35 b
**K fertilizer rate (kg ha**^**−1**^**)**						
150	1393.00 c	164.50 b	0.32 b	1.51 c	14,113.50 c	14,989.31 c
300	1479.67 a	176.17 a	0.35 a	1.60 a	14,754.17 a	15,905 04 a
450	1415.16 b	168.67 b	0.33 b	1.55 b	14,487.33 b	15,528.78 b
**N × K interaction**						
Control	1229.67 e	75.33 e	0.23 c	0.90 d	8142.02 e	10,214.56 e
CRU × KCl150	1434.09 bc	173.66 b	0.33 b	1.56 bc	14,521.72 bc	15,490.34 c
CRU × KCl300	1545.33 a	188.45 a	0.36 a	1.67 a	15,411.23 a	16,699.76 a
CRU × KCl450	1463.73 b	178.76 b	0.34 ab	1.61 ab	14,935.33 ab	16,039.72 b
Urea × KCl150	1352.01 d	155.33 d	0.31 b	1.46 c	13,705.49 d	14,488.36 d
Urea × KCl300	1414.21 c	164.98 c	0.34 ab	1.52 bc	14,097.34 cd	15,111.23 c
Urea × KCl450	1367.03 d	159.35 cd	0.32 b	1.49 c	14,039.76 cd	15,017.74 c
**Source of variance**						
N	< 0.0001	< 0.0001	0.0111	< 0.0001	< 0.0001	< 0.0001
K	< 0.0001	0.0013	0.0197	0.0003	0.0002	0.0004
N × K	0.063	0.3063	0.9734	0.1898	0.0321	0.0937

Means followed by different lowercase letters in the same column were significantly different based on analyses with ANOVAs followed by Duncan tests (*P* < 0.05).

*Control* no N and K fertilizer, *CRU* controlled-release urea, *KCl* potassium chloride.

### Yield

The dry Italian ryegrass yields of the CRU treatment increased continuously in four clipping periods in 2019 and 2020 (Table 6), those of the control treatment and common urea treatment decreased at the third clipping stage, and the difference was significant. In the whole growth period, under the CRU treatment conditions, there was no difference in the treatment with different amounts of KCl fertilizer, which indicated that the factors affecting the dry yields of Italian ryegrass were due more to N fertilizer than to K fertilizer. However, there was no significant N × K interaction effect on the yield. When clipping at the second stage, the highest dry yields of the CRU × KCl300 and CRU × KCl450 treatments were 1.75 g plant^−1^, which were 5.4% and 2.9% higher than that of the urea × KCl300 and the urea × KCl450 treatments, respectively, at the same K rate. During the third and fourth stages, the growth trend of the plants increased slowly, the demand for nutrients decreased, and the CRU slowly released N for plant growth. Thus, the CRU was more beneficial than common urea. In addition, K fertilizer had little influence on the yield. There was no significant N × K interaction effect on the dry yield (except at the fourth clipping).

**Table 6:** The dry yield of Italian ryegrass in 2019 and 2020.

Treatment	First clipping (g plant^−1^**)**	Second clipping (g plant^−1^**)**	Third clipping (g plant^−1^**)**	Fourth clipping (g plant^−1^**)**	Sum (g plant^−1^**)**	First clipping **(**g plant^**−**1^)	Second clipping (g plant^−1^**)**	Third clipping (g plant^−1^**)**	Fourth clipping (g plant^−1^**)**	Sum (g plant^−1^**)**
	2019					2020				
**N fertilizer type**										
CRU	1.24 a	1.62 a	1.67 a	0.96 a	5.48 a	1.03 a	1.74 a	1.91 a	1.01 a	5.68 a
Urea	1.16 b	1.49 b	1.56 b	0.86 b	5.07 b	0.94 b	1.57 a	1.61 b	0.72 b	4.85 b
**K fertilizer rate (kg ha**^**−1**^**)**										
150	1.18 b	1.52 b	1.58 b	0.88 b	5.16 b	1.03 a	1.74 a	1.91 a	1.01 a	5.68 a
300	1.22 1	1.57 a	1.64 a	0.92 a	5.35 a	0.94 b	1.57 a	1.61 b	0.72 b	4.85 b
450	1.18 b	1.57 a	1.64 a	0.93 a	5.32 a	1.03 a	1.74 a	1.91 a	1.01 a	5.68 a
**N × K interaction**										
Control	1.02 f.	1.33 e	1.30 e	0.73 e	4.38 f.	0.98 ab	1.44 ab	1.34 c	0.62 c	4.38 d
CRU × KCl150	1.23 b	1.58 b	1.63 b	0.93 b	5.37 c	1.02 a	1.71 a	1.95 a	0.92 b	5.61 ab
CRU × KCl300	1.27 a	1.63 a	1.69 a	0.98 a	5.57 a	1.02 a	1.75 a	1.79 ab	1.19 a	5.75 a
CRU × KCl450	1.20 bc	1.64 a	1.69 a	0.97 a	5.50 b	1.03 a	1.75 a	1.98 a	0.92 b	5.68 ab
Urea × KCl150	1.13 e	1.46 d	1.52 d	0.83 d	4.95 e	0.95 b	1.34 b	1.71 abc	0.60 c	4.59 cd
Urea × KCl300	1.17 cd	1.51 c	1.58 c	0.87 c	5.12 d	0.93 b	1.66 ab	1.67 abc	0.79 bc	5.06 bc
Urea × KCl450	1.16 de	1.51 c	1.59 c	0.88 c	5.14 d	0.96 ab	1.70 a	1.45 bc	0.78 bc	4.88 cd
**Source of variance**										
N	< 0.0001	< 0.0001	< 0.0001	< 0.0001	< 0.0001	0.0018	0.0868	0.0171	0.001	0.0013
K	0.0262	0.0002	0.0002	0.0018	< 0.0001	0.8091	0.1773	0.6016	0.0313	0.3924
N × K	0.0633	0.9029	0.5575	0.6343	0.0583	0.9421	0.2891	0.263	0.2286	0.7365

Means followed by different lowercase letters in the same column were significantly different based on analyses with ANOVAs followed by Duncan tests (*P* < 0.05).

*Control* no N and K fertilizer, *CRU* controlled-release urea, *KCl* potassium chloride.

### N and K use efficiency

The N fertilizer application significantly affected N uptake and N use efficiency (NUE), and the K fertilizer application significantly affected K uptake and K use efficiency (KUE) (Table 7). The N uptake and NUE of the CRU treatments were significantly higher than those of the urea treatments. In addition, K uptake increased with increasing KCl rate, but the KUE had the opposite trend. In total, the CRU × KCl300 treatment resulted in the highest nutrient uptake and use efficiency.

**Table 7:** The N uptake, K uptake, N use efficiency (NUE) and K use efficiency (KUE) of Italian ryegrass.

Treatment	N uptake (kg ha^−1^)	K uptake (kg ha^−1^)	NUE (%)	KUE (%)
**N fertilizer type**				
CRU	313.56 a	298.78 a	38.37 a	31.77 a
Urea	298.78 b	297.22 a	33.45 b	30.46 a
**K fertilizer rate (kg ha**^**−1**^**)**				
150	305.50 a	270.67 c	35.82 a	33.58 a
300	308.01 a	304.00 b	36.05 a	34.79 a
450	305.32 a	319.33 a	35.86 a	24.98 b
**N × K interaction**				
Control	202.33 c	212.61 d	–	–
CRU × KCl150	312.00 a	270.04 c	38.15 b	33.69 a
CRU × KCl300	315.67 a	306.33 ab	38.59 a	36.59 a
CRU × KCl450	313.22 b	320.09 a	38.36 ab	25.03 b
Urea × KCl150	299.12 b	271.33 c	33.49 c	33.46 a
Urea × KCl300	300.36 b	301.67 b	33.50 c	32.99 a
Urea × KCl450	297.11 b	318.64 a	33.35 c	24.93 b
**Source of variance**				
N	< 0.0001	0.6245	< 0.0001	0.2911
K	0.2975	< 0.0001	0.1275	0.0002
N × K	0.7215	0.7333	0.1572	0.417

Means followed by different lowercase letters in the same column were significantly different based on analyses with ANOVAs followed by Duncan tests (*P* < 0.05).

*Control* no N and K fertilizer, *CRU* controlled-release urea, *KCl* potassium chloride.

## Discussion

### Soil inorganic N and K form

The contents of soil inorganic N (NO_3_^−^-N and NH_4_^+^-N) in the 0–20 cm soil layer were greatly affected by fertilization and were related to the type of fertilizer, the times of fertilization and the amount of fertilization^27^. In the present study, the contents of nitrate N and ammonium N at depths of 0–20 cm were significantly affected by N fertilization, but no significant difference was found between the K fertilization applications. At the beginning, urea rapidly dissolved and released substantial N, while the CRU initially released less N; thus, the soil inorganic N content of the urea treatment was higher than that of the CRU treatment at the first clipping stage. However, due to the continuous release of N from the CRU (Fig. 1), the contents of NO_3_^−^-N and NH_4_^+^-N increased significantly from the second clipping stage to the fourth clipping stage compared with that in the urea treatment (Fig. 2). The blend application of CRU mixed with urea significantly increased the soil inorganic N than the normal urea, especial in the later growth stage^27^. The effect of the KCl rate on soil inorganic N was not obvious during the whole growth stage of Italian ryegrass.

The application of K fertilizer significantly affects the content of soil K and the form of soil K^16,28^. In the present study, K application significantly increased the contents of soil available K, water-soluble K, exchangeable K and non-exchangeable K, similar to other research results^29^. However, quick-acting fertilizers such as KCl are easily converted into non-exchangeable K, which leads to a decrease in soil K availability^30^. In total, the contents of soil available K, water-soluble K, exchangeable K and non-exchangeable K increased with increasing KCl rate (Fig. 3). The content of soil available K and water-soluble K had significant positive correlation, where similar presence also found in exchangeable K and non-exchangeable K (Table 8). The water-soluble K contributes the most to NO_3_^−^-N and the available K got similar effects on NH_4_^+^-N. In addition, the contents of soil available K in the KCl300 and KCl450 treatments were similar. Thus, a moderate amount of K fertilizer could maintain a high K content in the soil, which would supply enough K nutrition for Italian ryegrass^31^. Besides, we found the interaction effect of the N × K on soil available K or inorganic N was not obvious during the whole growth stage of Italian ryegrass. The interaction effect of the N and K fertilization on soil K and N was not obvious in a cotton field^25^.

**Table 8:** Correlations between different forms of soil K and N.

Correlation coefficient	Available K	Water soluble K	Exchangeable K	Non-exchangeable K	NO_3_^−^-N	NH_4_^+^-N
Available K	1					
Water soluble K	0.8733**	1				
Exchangeable K	0.6775**	0.2333*	1			
Non-exchangeable K	0.8455**	0.8248**	0.4423**	1		
NO_3_^−^-N	0.7592**	0.8370**	0.2516*	0.5092**	1	
NH_4_^+^-N	0.7919**	0.7695**	0.4189**	0.5579**	0.8917**	1

*P < 0.05; **P < 0.01.

### Growth index

To understand the growth of crops over time, it is necessary to determine their nutritional status. The traditional determination of leaf colour is to detect the chlorophyll content, which has high accuracy, but it takes considerable work and time^32^. Plant height, chlorophyll, leaf area and photosynthetic index are important parameters to characterize crop photosynthetic production capacity, crop growth and nutritional status^33^. The results showed that in comparison to the CRU treatment, the urea treatment significantly reduced the plant height, SPAD, leaf area and photosynthetic index, which might have led to plant senescence.

In addition, the application of KCl significantly increased the chlorophyll content in Italian ryegrass. Within a certain range of KCl applications, the chlorophyll content increased with increasing KCl applications, but beyond this threshold, the effect of KCl was weakened, thus affecting the ornamental value of Italian ryegrass. This outcome may have been due to the decrease in assimilative capacity, enzyme content and enzyme activity caused by the lack or excess of K, further leading to the decrease in leaf area and photosynthesis and thus affecting photosynthesis^34^. Generally, the CRU × KCl300 treatment improved the leaf photosynthesis of Italian ryegrass.

Roots play an important role in crop growth and yield formation^35^. In the present study, the application of the CRU and moderate KCl rate significantly increased root length, surface areas and the numbers of tips and branches than other treatments, and there was significant N × K interaction effect on the the numbers of tips. These results suggested that suitable N and K fertilizer application can enhance root growth and thereby increase the uptake of nutrients^36^. Totally, there was no significant N × K interaction effect on the growth index. A positive correlation between the duration of reproductive growth and the appropriate amount of N or K application, but the interaction effect of N × K was not obvious^26^.

### Yield and fertilizer use efficiency

N and K are the key factors affecting the yield of Italian ryegrass. Under sufficient N, the leaves of Italian ryegrass are thick green and have strong growth and a high yield of fresh grass, and under N deficiency, the leaves of Italian ryegrass are yellow and have poor growth^37^.

The results showed that in comparison to those of the urea treatment and the control treatment, the dry yields of the CRU treatment were highest and increased by 20.5–53.2% (Table 6). Studies have also shown that CRU effectively promotes the ability of photosynthesis to produce organic matter and then increase plant yield^38,39^. In this study, the second and third clipping stages were the period of high yield of Italian ryegrass and the period of high yield of CRU. It highlighted that N uptake of Italian ryegrass in the first growing stages is lower and this can be functional to the presented pattern of N release in soil^11^. In the present study, the yield of the CRU treatment was higher than that of the common urea treatment in the four clipping periods, especially in the middle and later stages of Italian ryegrass growth, which occurred because the CRU slowly released N and met the growth demand of Italian ryegrass. In general, the N fertilizer type markedly affected the dry yield, but the application amount of K fertilizer had little effect on the yield. Moreover, no significant N × K interaction effect was found in the present study, which was different^24^. Hence, it is necessary and important to carry out further field experiments on the accurate rate and timing of N and K. Through a comparison, the yield of the CRU × KCl450 treatment was the highest in the early stage, but considering the later stage yield, fertilizer utilization rate, economic benefits and other factors, the whole growth period of the CRU × KCl300 treatment was the most suitable treatment combination of all treatments, which improved the yield of Italian ryegrass.

There are many parameters to describe the efficiency of fertilizer utilization. The key to improving the efficiency of fertilizer utilization is nutrient absorption^40^. In this study, NUE and KUE were used. Regardless of the amount of KCl applied, the NUE of the CRU treatment was significantly higher than that of the urea treatment, which might have been due to high N absorption, and similar to the results^18^. In addition, the KCl rate had a significant effect on K absorption and KUE (Table 7). The KUE values of the KCl300 treatment were higher than those of the KCl150 and KCl450 treatments. Therefore, the CRU × KCl300 treatment could improve the NUE and KUE of Italian ryegrass. Besides, the increase nutrient-use efficiency could also minimize unfavorable effects on the environment, mainly leaching^11^.

Overall, the N fertilizer type and K fertilizer rate had significant effects on Italian ryegrass growth, yield and soil fertility, but there was no significant N × K interaction effect. The CRU released N slowly, which was consistent with the N demand of Italian ryegrass during the whole growth and developmental period, simplifying the cultivation technology and reduced the soil N/K leaching. This study found that the amount of K fertilizer had no significant effect on the growth of Italian ryegrass in the early stage, but in the middle and late stages of ryegrass growth, the CRU × KCl300 treatment improved plant SPAD and root morphology, delayed senescence of Italian ryegrass, and significantly increased the yield and fertilizer use efficiency. Hence, the CRU × KCl300 treatment is recommended as the best fertilization combination for Italian ryegrass, and this recommendation can serve technical support for Italian ryegrass production and fertilization.

## Materials and methods

### Experimental materials

The two-year experiment (2018–2019) was conducted at the Yinan County experimental base, Linyi City, Shandong Province, China (N 35°48′33″; E 118°26′45″). The climate is a temperate monsoon climate, and the precipitation is concentrated from July to September. The contents of sand, silt and clay in the soil are 547.32, 228.63 and 216.03 g kg^−1^, respectively, which are classified as sandy loam. The two-year field experiment was carried out at the experimental base of the College of Agriculture and Forestry Science, Linyi University, Linyi city, Shandong Province (35°06'N, 118°17'E) in 2019 and 2020, and this site has a continental climate typical of temperate monsoon areas. The temperature and relative humidity in the greenhouse were 30 ± 5 °C and 45 ± 5% mm, respectively.

### Soil type and analysis

The field soil is sandy loam, which is classified as Typic Hapludalf according to the USDA classification^41^. The basic soil properties are listed in Table 9.

**Table 9:** Part properties of tested soil before ryegrass planting in 2019.

pH value (2.5:1)	Organic matter (g kg^−1^)	Total N (g kg^−1^)	NO_3_^—^N (mg kg^−1^)	NH_4_^+^-N (mg kg^−1^)	Available P (mg kg^−1^)	Available K (mg kg^−1^)
6.52	6.88	0.73	46.09	31.22	39.82	182.08

### Fertilizer material

**Figure 4:**
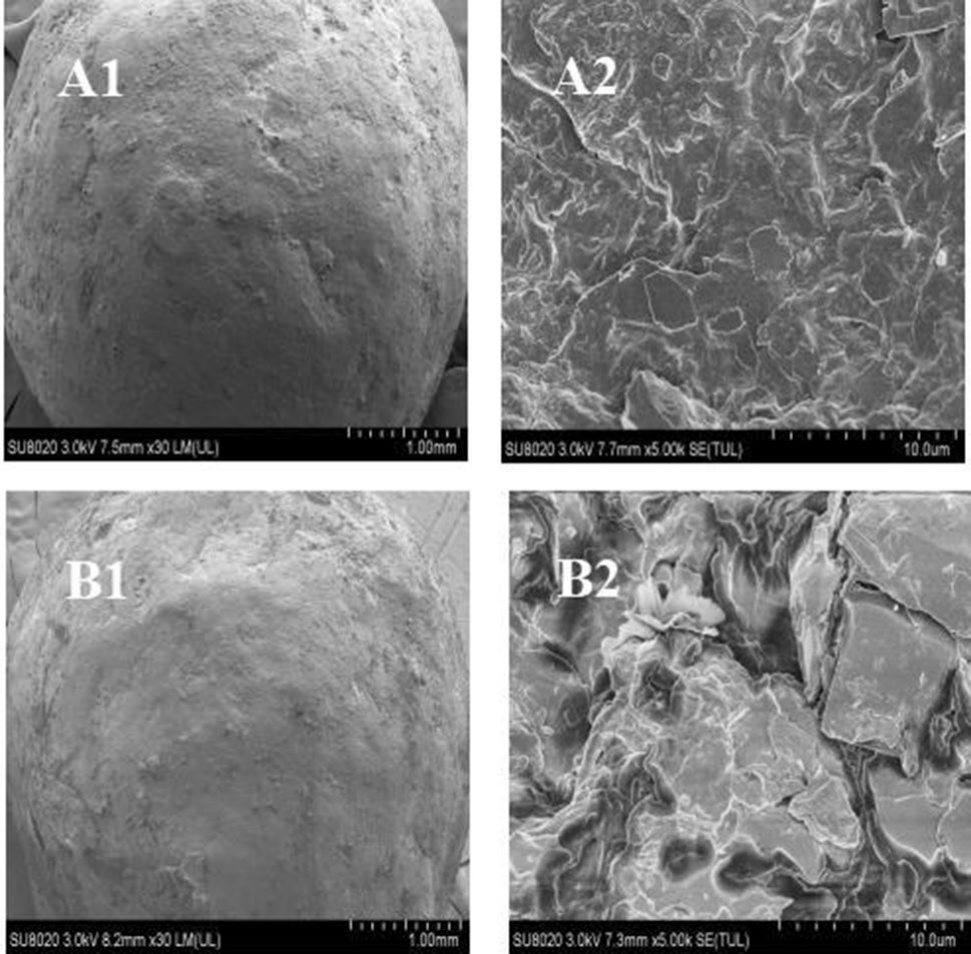
Scanning electron micrographs of (A1-1 mm and A2-10 µm) CRU before release and (B1-1 mm and B2-10 µm) CRU after release.

**Figure 5:**
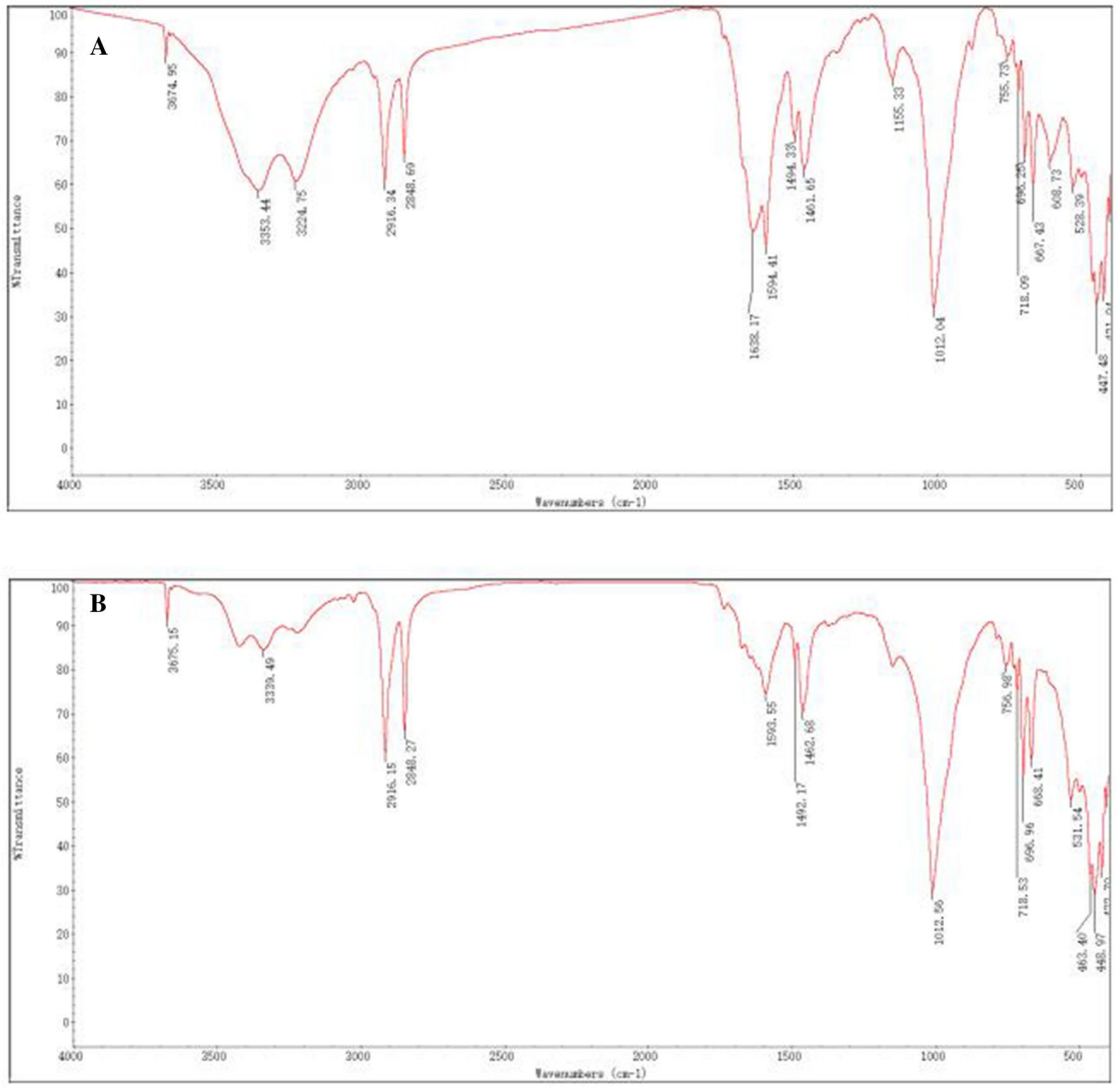
FTIR spectra of (**A**) CRU before release and (**B**) CRU after release.

The fertilizers used included controlled-release urea (CRU) and ordinary fertilizers. The CRU (containing N 43%, release period was almost 3 months in distilled water at 25 °C) fertilizer was produced by the State Key Laboratory of Nutrition Resources Integrated Utilization, China. The CRU fertilizer was formulated as round particles with a regular shape and smooth surface, and it was coated with an epoxy resin (low curing shrinkage, strong adhesion and chemical resistance). The polyurethane epoxy resin synthesized from polyethylene glycol and hydroxyl terminated polycaprolactone has good biodegradability, which the SEM micrographs (Fig. 4) and FTIR spectra (Fig. 5) revealed changes in the characterization results of CRU with nutrient release after the incorporation of K salts. Surface of the shell became micro-holes and rough after release. Indicating that the coating material will degrade quickly without harming the environment. The ordinary fertilizers included urea (containing N 46%), calcium superphosphate (containing P_2_O_5_ 14%), and potassium chloride (KCl) (containing K_2_O 60%), which were provided by Jinyimeng Group Co., Ltd., and Kingenta Ecological Engineering Group Co., Ltd., China.

### Experimental design

A split-plot design with three replications was used for the experiment. Specifically, the N types (CRU and urea) were the main plots, potassium chloride (KCl) rates (150, 300 and 450 kg ha^−1^) were the subplots, and no N and K fertilization were the control. Each plot received a basal application of 300 kg ha^−1^ N and 200 kg ha^−1^ P_2_O_5_. All fertilizers (except common urea) were applied once with hands before sowing seeds. In particular, the urea applied twice in total, with 60% before sowing seeds, and 40% after the second clipping stage. The Italian ryegrass was sown on April 4, 2019 and April 7, 2020. CRU (10 g) was weighed and placed into the mesh bag (8 cm width and 10 cm length), the bags were sealed, and this process was repeated 18 times. The tested forage was ‘Bluesign’ Italian ryegrass, which is produced by Suqian Chengzhiyang Seed Industry Co., Ltd., China.

### CRU release characteristics

The release of the CRU in the soil was determined by the burying bag weighing method^17^. Similarly, 10 g of CRU particles was put into the mesh bag (8 cm width and 10 cm length) and buried in a cement tank with a depth of 15–20 cm during fertilization. The net bags were collected on the 10th, 20th, 30th, 60th, 90th, and 120th days after burial in the soil. Then, three bags were collected each time, washed and dried to constant weight at 60 °C, and the N release rate was calculated according to the weight of the remaining fertilizer particles.

### Soil sampling and measurement

The plant and soil samples were determined in 2020. The 0–20 cm soil samples were collected by a 5-point sampling method (2 sampling points in the fertilizer row, 3 sampling points in the the plant row). The contents of NO_3_^−^-N and NH_4_^+^-N in fresh soil (0.01 mol L^−1^ CaCl_2_ extraction) were determined immediately using an AA3 continuous flow analyser (Bran-Luebbe, Norderstedt, Germany). The remaining soil was dried by naturally existing air, ground through 2 mm and 0.25 mm sieves. The organic matter was determined by the K dichromate external heating method. The soil total N was determined by the semi micro Kelvin method. The available phosphorus was determined by the pH 8.5, 0.5 mol L^−1^ NaHCO_3_ extraction, molybdenum blue colorimetry. The available K content was determined by the 1 mol L^−1^ NH_4_OAc extraction, flame photometer method^27^.

### Leaf sampling and analysis

The leaf area of Italian ryegrass was measured by a leaf area metre (Yaxin-1241, Yaxinliyi, China). The SPAD value (chlorophyll relative value) were used as a proxy of the chlorophyll content of leaves (SPAD-502, Minolta, Japan). The Li-6400 portable photosynthetic apparatus (LI-COR, Lincoln, NE, USA) was also used for the determination. The leaf photosynthetic indicators were measured at 9:00–10:00 a.m. Selecting sunny and cloudless weather included the net photosynthetic rate (*P*_n_), stomatal conductance (*G*_s_), intercellular carbon dioxide concentration (*C*_*i*_) and transpiration rate (*T*_r_) before the second clipping.

### Root sampling and analysis

The root samples were scanned with a rhizome scanner (WinRhizo STD 4800, CAN) to determine the root volume, total length, diameter, surface area, number of tips and branches^42^.

### Harvest measurement

After measuring the physiological indexes of the plant, the grass was clipped with scissors for each treatment, and the height of the stubble was the same as that of the ground. Then, the clipped grass was sealed in the file bag according to the treatment label, placed in the oven at 105 °C for 30 min, and dried at 65 °C for 72 h, and then, the dry weight of the yield was recorded. Finally, the N and K contents of the plants were analysed. The plant total N contents were determined by digestion with H_2_SO_4_-H_2_O using the micro-Kjeldahl method, and the K content of the plant was digested by H_2_SO_4_-H_2_O and determined by a flame photometer^27^. The N and K uptake were calculated according to the N and K content and dry mass weight. The N use efficiency (NUE) and K use efficiency (KUE) were calculated^43^.

### Statistical analyses

Microsoft Excel 2010 was employed for data processing and figure drawing. Data was subjected to analysis of variance and mean separation tests as a split-plot factorial design with three replications. Concretely, the data were analyzed using the Statistical Analysis System version 9.2 (SAS Institute Cary, NC, 2010) with a two-way ANOVA at a significance level of 0.05, with N type and K rate as the independent variables. Two-way analyses of variance (ANOVAs) were performed to determine the effects of N, K and their interactions on the leaf area, leaf SPAD, leaf photosynthesis chlorophyll parameters, root morphology, yield and nutrient uptake of Italian ryegrass. One-way analyses of variance were performed to test for significant differences between treatments of NH_4_^+^-N, NO_3_^−^-N, and soil K informs. A Duncan multiple range test was carried out to determine if significant (p < 0.05) differences occurred between individual treatments^44^.

### Ethics approval

Experimental research and field studies on plants comply with relevant institutional, national, and international guidelines and legislation.

## Data Availability

The datasets used and analysed during the current study available from the corresponding author on reasonable request.

## References

[1] Liu M, Dries L, Heijman W, Huang J, Zhu X, Hu Y, Chen H (2018). The impact of ecological construction programs on grassland conservation in Inner Mongolia, China. Land Degrad. Dev..

[2] Binder S, Isbell F, Polasky S, Catford JA, Tilman D (2018). Grassland biodiversity can pay. Proc. Nalt. Acad. Sci. USA.

[3] Pinto AG, Barreto MT, Valle TAD, Dornelles RDR, Comassetto DDS, Faleiro EA, Rodriguse CR, de Azevedo EB (2022). Forage production, morphological, and chemical composition of diploid and tetraploid cultivars of italian ryegrass in hydromorphic soils. N. Z. J. Agric. Res..

[4] Svatos KB, Abbott LK (2019). Dairy soil bacterial responses to nitrogen application in simulated Italian ryegrass and white clover pasture. J. Dairy Sci..

[5] Fan X, Kawamura K, Xuan TD, Yuba N, Lim J, Yoshitoshi R, Obitsu T (2018). Low-cost visible and near-infrared camera on an unmanned aerial vehicle for assessing the herbage biomass and leaf area index in an Italian ryegrass field. Grass. Sci..

[6] Alves dos Santos, J., da Fonseca, A. F., Barth, G. & Zardo Filho, R. Silage maize quality in different uses of Italian ryegrass and soil management methods after liming. *Arch. Agron. Soil Sci.* **64**(2), 173–184. 10.1080/03650340.2017.1338832 (2018).

[7] He HB, Li WX, Zhang YW, Cheng JK, Jia XY, Li S, Xin GR (2020). Effects of Italian ryegrass residues as green manure on soil properties and bacterial communities under an Italian ryegrass (*Lolium multiflorum* L.)-rice (*Oryza sativa* L.) rotation. Soil Till Res..

[8] Sandaa P, Lobos IA, Pavez PB, Moscoso CJ (2021). Nitrogen nutrition index and forage yield explain nitrogen utilization efficiency in hybrid ryegrasses under different nitrogen availabilities. Field Crops Res..

[9] Woods RR, Cameron KC, Edwards GR, Di HJ, Clough TJ (2018). Reducing nitrogen leaching losses in grazed dairy systems using an Italian ryegrass-plantain-white clover forage mix. Grass Forage Sci..

[10] Cavalli D, Cabassi G, Borrelli L, Geromel G, Bechini L, Degano L, Gallina PM (2016). Nitrogen fertilizer replacement value of undigested liquid cattle manure and digestates. Eur. J. Agron..

[11] Masoni A, Mariotti M, Ercoli L, Pampana S, Arduini I (2015). Nitrate leaching from forage legume crops and residual effect on italian ryegrass. Agrochim. Pisa.

[12] Wang H, Wu L, Cheng M, Fan J, Zhang F, Zou Y, Wang X (2018). Coupling effects of water and fertilizer on yield, water and fertilizer use efficiency of drip-fertigated cotton in northern Xinjiang, China. Field Crop Res..

[13] Ata-Ul-Karim ST, Liu X, Lu Z, Zheng H, Cao W, Zhu Y (2017). Estimation of nitrogen fertilizer requirement for rice crop using critical nitrogen dilution curve. Field Crop Res..

[14] Martin K, Edwards G, Bryant R, Hodge M, Moir J, Chapman D, Cameron K (2017). Herbage dry-matter yield and nitrogen concentration of grass, legume and herb species grown at different nitrogen-fertiliser rates under irrigation. Anim. Prod. Sci..

[15] Geng JB, Ma Q, Chen JQ, Zhang M, Li CL, Yang YC, Yang XY, Liu ZG (2016). Effects of polymer coated urea and sulfur fertilization on yield, nitrogen use efficiency and leaf senescence of cotton. Field Crop Res..

[16] Li, J., Niu, L., Zhang, Q., Di, H. & Hao, J. Impacts of long-term lack of potassium fertilization on different forms of soil potassium and crop yields on the North China Plains. *J. Soil Sediment.* **17**(6), 1607–1617. 10.1007/s11368-017-1658-8 (2017)

[17] Liu J, Yang Y, Gao B, Li YC, Xie J (2019). Bio-based elastic polyurethane for controlled-release urea fertilizer: Fabrication, properties, swelling and nitrogen release characteristics. J. Clean. Prod..

[18] Cordeiro, C., Rodrigues, D. R. & Echer, F. R. Cover crops and controlled-release urea decrease need for mineral nitrogen fertilizer for cotton in sandy soil. *Field Crops Res.* **276**, 108387. 10.1016/j.fcr.2021.108387 (2022).

[19] McDonnell RP, Staines MV, Bolland MDA (2018). Determining the critical plant test potassium concentration for annual and Italian ryegrass on dairy pastures in south-western Australia. Grass Forage Sci..

[20] Hasanuzzaman M, Bhuyan MHM, Nahar K, Hossain M, Mahmud JA, Hossen M, Fujita M (2018). Potassium: A vital regulator of plant responses and tolerance to abiotic stresses. Agronomy.

[21] Oliveira, E. M. D., Oliveira Filho, J. D. C., Oliveira, R. A. D., Oliveira, R. M. D. & Cecon, P. R. Determination of xaraés grass quality submitted to irrigation water levels and nitrogen and potassium doses. *Eng. Agr-Jaboticabal.* **7**(1), 64–74. 10.1590/1809-4430-eng.agric (2017).

[22] Kokulan, V., Wadu, M., Akinremi, O. O. & Buckley, K. E. Nitrogen and phosphorus distribution in plant, soil, and leachate as affected by liquid hog manure and chemical fertilizers. *Can. J. Soil Sci*. 10.1139/CJSS-2020-0080 (2021).

[23] Snyder GH, Cisar JL (2000). Nitrogen/potassium fertilization ratios for bermudagrass turf. Crop Sci..

[24] Yang XY, Geng JB, Li CL, Zhang M, Chen BC, Tian XF, Zheng WK, Liu ZG, Wang C (2016). Combined application of polymer coated potassium chloride and urea improved fertilizer use efficiencies, yield and leaf photosynthesis of cotton on saline soil. Field Crops Res..

[25] Yang XY, Geng JB, Li CL, Zhang M, Tian XF (2016). Cumulative release characteristics of controlled-release nitrogen and potassium fertilizers and their effects on soil fertility, and cotton growth. Sci. Rep..

[26] Dong HZ, Kong XQ, Li WJ, Tang W, Zhang DM (2010). Effects of plant density and nitrogen and potassium fertilization on cotton yield and uptake of major nutrients in two fifields with varying fertility. Field Crops Res..

[27] Zheng W, Sui C, Liu Z, Geng J, Tian X, Yang X, Zhang M (2016). Long-term effects of controlled-release urea on crop yields and soil fertility under wheat-corn double cropping systems. Agron. J..

[28] Yang XY, Li CL, Zhang Q, Liu ZG, Geng JB, Zhang M (2017). Effects of polymer-coated potassium chloride on cotton yield, leaf senescence and soil potassium. Field Crops Res..

[29] Kurbah I, Dixit SP (2019). Soil potassium fractions as influenced by integrated fertilizer application based on soil test crop response under maize-wheat cropping systems in Acid Alfisol. Int. J. Econ. Plant..

[30] Chen J, Guo Z, Chen H, Yang X, Geng J (2020). Effects of different potassium fertilizer types and dosages on cotton yield, soil available potassium and leaf photosynthesis. Arch. Agron. Soil Sci..

[31] Jiménez-Calderón JD, Martínez-Fernández A, Benaouda M, Vicente F (2018). A winter intercrop of faba bean and rapeseed for silage as a substitute for Italian ryegrass in rotation with maize. Arch. Agron. Soil Sci..

[32] Croft H, Chen JM, Luo X, Bartlett P, Chen B, Staebler RM (2017). Leaf chlorophyll content as a proxy for leaf photosynthetic capacity. Glob. Change Biol..

[33] Kuan-Hung LIN, Chun-Wei WU, Chang YS (2019). Applying dickson quality index, chlorophyll fluorescence, and leaf area index for assessing plant quality of Pentas lanceolata. Not. Bot. Hortic. Agrobo..

[34] Lu Z, Pan Y, Hu W, Cong R, Ren T, Guo S, Lu J (2017). The photosynthetic and structural differences between leaves and siliques of Brassica napus exposed to potassium deficiency. BMC Plant Biol..

[35] Inagakia, T. M., JCDM Sá, Tormena, C. A., Dranski, A. & Silvae, L. P. Mechanical and biological chiseling impacts on soil organic c stocks, root growth, and crop yield in a long-term no-till system. *Soil Till. Res.* **211**(1), 104993. 10.1016/j.still.2021.104993 (2021).

[36] Enriquez-Hidalgo, D., Gilliland, T. J., Egan, M. & Hennessy, D. Production and quality benefits of white clover inclusion into ryegrass swards at different nitrogen fertilizer rates. *J. Agric. Sci.*10.1017/S0021859618000370 (2018).

[37] Vleugels T, Rijckaert G, Gislum R (2017). Seed yield response to N fertilization and potential of proximal sensing in Italian ryegrass seed crops. Field Crop Res..

[38] Van Eerd LL, Turnbull JJD, Bakker CJ, Vyn RJ, McKeown AW, Westerveld SM (2017). Comparing soluble to controlled-release nitrogen fertilizers: storage cabbage yield, profit margins, and N use efficiency. Can. J. Plant Sci..

[39] Miyatake M, Ohyama T, Yokoyama T, Sugihara S, Motobayashi T, Kamiya T, Ohkama-Ohtsu N (2019). Effects of deep placement of controlled-release nitrogen fertilizer on soybean growth and yield under sulfur deficiency. Soil Sci. Plant Nutr..

[40] Xue X, Mai W, Zhao Z, Zhang K, Tian C (2017). Optimized nitrogen fertilizer application enhances absorption of soil nitrogen and yield of castor with drip irrigation under mulch film. Ind. Crop Prod..

[41] Soil Survey Staff. Soil taxonomy. In: Soil Survey Staff (Ed.), a basic system of soil classification for making and interpreting Soil Surveys, 2nd U.S. Gov. Print. Office, Washington, DC. pp. 163–167 (1999).

[42] Naeem, I., Asif, T., Zhang, T. Y., Guan, Y., Wu, X. F., Tariq, H. & Wang, D. L. Mixing effects of three Eurasian plants on root decomposition in the existence of living plant community in a meadow steppe. *Sci. Total Environ.*10.1016/j.scitotenv.2021.151400 (2022). 10.1016/j.scitotenv.2021.151400 34742802

[43] Rietra RP, Heinen M, Dimkpa CO, Bindraban PS (2017). Effects of nutrient antagonism and synergism on yield and fertilizer use efficiency. Commun. Soil Sci. Plant.

[44] Tang Q, Feng M (2002). Practical Statistics and DPS Data Processing System.

